# Etude de la flore bactérienne contaminant les téléphones mobiles avant et après la désinfection: comparaison entre les professionnels soignants de l'hôpital militaire d'instruction Mohammed V de Rabat et les témoins

**DOI:** 10.11604/pamj.2015.22.326.7292

**Published:** 2015-12-02

**Authors:** Jean Uwingabiye, Wafaa Moustanfii, Meryem Chadli, Yassine Sekhsokh

**Affiliations:** 1 Laboratoire de Recherche et de Biosécurité P3, Hôpital Militaire d'Instruction Mohammed V, Faculté de Médecine et de Pharmacie de Rabat, Université Mohammed V Rabat, Rabat, Maroc

**Keywords:** Téléphone mobile, contamination bactérienne, personnels de santé, solution hydroalcoolique, décontamination, Mobile phone, bacterial contamination, health personnel, hand sanitizer, decontamination

## Abstract

**Introduction:**

L'objectif de notre travail était évaluer la contamination microbienne des téléphones mobiles utilisés par les personnels soignants des différents services de l'hôpital militaire d'instructions Mohammed V de Rabat et la comparer à celui d'une population témoin et aussi démontrer l'efficacité des solutions hydroalcoolique dans la désinfection de ces téléphones mobiles.

**Méthodes:**

Il s'agit d'une étude descriptive transversale réalisée sur une période de 9 mois entre septembre 2010 et juin 2011, dans le service de bactériologie de l'hôpital militaire d'Instruction Mohammed V.

**Résultats:**

L’étude bactériologique a été faite sur 240 téléphones mobiles dont 50% provenaient de personnels de sante. Le taux de contamination bactérienne de tous les téléphones mobiles était de 100%. Les cultures des bactéries isolées au niveau des téléphones mobiles du personnel médical étaient plus polymorphes que celles de la population témoin (p=0,028). Parmi 437 bactéries isolées: 223(51%) provenaient de téléphones de personnels de santé et 214(49%) de téléphones de la population témoin avec une différence qui n’était pas statistiquement significative(p>0,05) sauf pour les isolats de *Staphylocoque à coagulase négative* et *Staphylococcus aureus*. Les bactéries isolées étaient représentées par: *Staphylocoque à coagulase* (57,7%), *Staphylococcus aureus* (18,1%), *Corynebacterium sp* (18,8%), *Bacillus sp* (2,3%) et autres (2,2%). La différence entre la prévalence des bactéries isolées selon les services et les fonctions des personnels de santé n’était pas statistiquement significative (p>0,05). La désinfection des téléphones portables par la solution hydroalcoolique a réduit à 99,5% le nombre des colonies.

**Conclusion:**

Ce travail montre que les téléphones portables pourraient jouer un rôle dans la transmission des infections nosocomiales et communautaires. Dans le cadre de prévention de ces risques, il faut sensibiliser les utilisateurs des téléphones mobiles l'importance du lavage des mains et l'utilisation des solutions hydro alcoolique pour désinfecter aussi bien les téléphones portables que les mains.

## Introduction

Les téléphones mobiles des personnels de la santé constituent un réservoir de bactéries [1, 2] et lors de chaque appel téléphonique, des téléphones mobiles sont en contact étroit avec les régions fortement contaminées du corps humain: les mains, la bouche, le nez et les oreilles. Ce sont des objets personnels utilisés aussi bien à l'intérieur des centres hospitaliers qu’à l'extérieur et dont la saleté provient de gestes simples, ils peuvent être a l'origine d'infections nosocomiales et communautaires [1, 2]. La recherche a montré qu'il pourrait constituer un risque sanitaire majeur de transmission des bactéries multi résistantes dans les établissements de soins de santé qui peuvent conduire à des infections graves associé à une forte morbidité, une mortalité élevé et à un surcoût médical supplémentaire [2]. Certaines études ont examiné la contamination microbienne des téléphones mobiles et le taux de contamination bactérienne des téléphones mobiles des personnels de la santé variait de 32% à 97,8% [2–4]. A notre connaissance, aucune étude concernant l’étude bactériologique des téléphones mobiles n'est réalisée au Maroc. Cette étude visait à évaluer la contamination microbienne des téléphones mobiles utilisés par les personnels soignants des différents services de l'hôpital militaire d'instructions Mohammed V de Rabat (HMIMV) et la comparer à celui d'une population témoin et aussi démontrer l'efficacité des solutions hydro alcoolique dans la désinfection de ces téléphones mobiles.

## Méthodes

Il s'agit d'une étude transversale de 9 mois, réalisée entre septembre 2010 et juin 2011, en collaboration entre le service de Bactériologie et les différents services de l'HMIMV qui a une capacité hospitalière de plus de 700 lits et qui comporte toutes les spécialités médicales et chirurgicales. Le choix des services et des sujets de l’étude a visé toutes les disciplines: médicales, chirurgicales, réanimation et laboratoire. L’échantillonnage était fait par volontariat d'une dizaine du personnel soignant par service, en tenant compte que l’échantillon soit représentatif en respectant la variabilité des personnels médicaux (médecins et pharmaciens), paramédicaux (infirmier, technicien, aides-soignants, secrétaire, ingénieur). Sont exclus de notre étude les services de l'HMIMV n'exerçant pas une activité de soin: logistiques, transport, services administratifs et autres. La population témoin était choisie d'une population n'exerçant pas dans le domaine de la santé. Les témoins ont été sélectionnés parmi les étudiants de l'Institut Spécialisé de Technologie Appliquée et de nouvelles technologies de l'information et de la communication (ISTA NTIC) de Hay Riad à Rabat. Chaque candidat a rempli une fiche d'exploitation réunissant les données concernant la fonction, services médicaux, matière du couvercle de téléphone portable et le type lavage des mains. Le prélèvement par écouvillonnage de chaque téléphone mobile était effectué avant et après désinfection par une solution hydro alcoolique (Aniosgel 85 NPC^®^) à base de l’éthanol (750 mg/g ou 755 ml/l) pendant 30 secondes. Ensuite l'ensemencement a été fait directement sur un milieu bromocrisol pourpre et sur un milieu Chapman. Les boites étaient acheminées rapidement au laboratoire de bactériologie de l'HMIMV. L'incubation était fait dans l’étuve à 37^°^c, prolongée jusqu’à 48 heures quand les colonies poussées à 24 heures étaient fines et ne permettant pas une bonne exploitation. L'identification des bactéries a été réalisée en se basant sur les caractères culturaux, morphologiques et biochimiques (galerie API, bio- Mérieux SA, Marcy-L’étoile, France). Les analyses statistiques étaient faites en utilisant le logiciel Statistics SPSS (Statistical Package for the Social Sciences) 17,0 et Epi-info version 7. Le test du chi-carré ou test exact de Fisher était utilisé pour comparer des pourcentages des isolats entre les personnels soignants et la population témoin et Le test t de Student a été utilisé pour la comparaison des moyennes. Les valeurs de p inférieur à 0,05 étaient considérée comme significatives.

## Résultats

**Figure 1:**
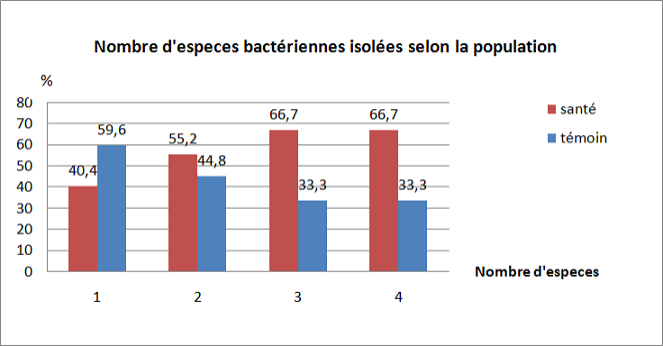
Pourcentage de cultures monomorphes et polymorphes entre les deux populations (p = 0,028)

L’étude bactériologique a été faite sur 240 téléphones mobiles dont 50% (120 /240) provenaient de personnels de santé. Le taux de contamination bactérienne de tous les téléphones mobiles était de 100%. Chez les personnels de santé, le nombre médian de colonies était 10 UFC (Unité formant colonie) avec un intervalle intervaquartile de [4-30] et chez les témoins, le nombre médian de colonies était de 16 UFC avec un intervalle intervaquartile de [6-30] (p = 0,052). La différence de caractère monomorphe ou polymorphe des cultures bactérienne entre les deux populations étaient statistiquement significative (p = 0,028); la culture bactérienne des téléphones des personnels de santé étaient plus polymorphe que celle de la population témoin (Figure 1). Parmi 437 bactéries isolées: 223(51%) provenaient de téléphones de personnels de santé et 214(49%) de téléphones de la population témoin avec une différence qui n’était pas statistiquement significative(p > 0,05) sauf pour les isolats de *Staphylocoque à coagulase négative* (SCN) et *Staphylococcus aureus*. Les bactéries isolées étaient représentées par: SCN (50,7% pour le personnel, 65% pour le témoin), S. aureus (18,1% pour le personnel; 22% pour le témoin), *Corynebacterium sp* (18,8%) pour le personnel; 20,2% pour le témoin);*Bacillus sp* (3,1% pour le personnel; 1,4% pour le témoin) et autres (2,2% pour le personnels; 2,4% pour le témoin) (Tableau 1). La différence d'isolement des bactéries selon la nature du couvercle entre les deux populations était statistiquement significative (p < 0,05) sauf pour les couvercles mixtes(métallique +plastique), ces bactéries étaient fréquemment isolées de téléphones dont le couvercle était plastiques (304/437 = 69,6%) suivi de ceux de couvercle métallique(123/437 = 28,1%) et ceux de couvercle métallique + plastique(6/437 = 2,8%) (Tableau 1).

**Tableau 1:** Répartition des bactéries isolées des téléphones mobiles des personnels de santé et de la population témoin

	Total N = 439	Personnels N = 223	Témoins N = 214	*p*
**Germes isolés**^+^				
**Cocci Gram positif**	**335(76,7)**	**166(74,5)**	**169(82,2)**	**0,23**
*SCN*	252(57,7)	113(50,7)	139(65)	0,002
*S aureus*	79(18,1)	49(22)	30(17,2)	0,03
*Streptoccocus sp*	4(0,9)	4(1,8)	-	-
**Bacilles Gram positif**	**92(21,1)**	**52(23,3)**	**40(15,4)**	**0,23**
*Corynebacterium sp*	82(18,8)	45(20,2)	37(14)	0,43
*Bacillus sp*	10(2,3)	7(3,1)	3(1,4)	0,34
**Bacilles Gram négatif**	**6(1,3)**	**3(1,3)**	**3(1,5)**	**0,95**
*E coli*	3(0,7)	2(0,9	1(0,5)	1
*Klebsiela sp*	1(0,2	-	1(0,5)	-
*Pantoea spp*	1(0,2)	1(0,4)	-	-
*Acenitobacter sp*	1(0,2)	-	1(0,5)	-
**Levures**	**4(0,9)**	**2(0,9)**	**2(0,9**	**0,96**
*Candida sp*	4(0,9)	2(0,9)	2(0,9	1
**Matière du téléphone**^+^				
Métallique	123(28,1)	87(39)	36(16,8)	<10^−5^
Plastique	304(69,6)	132(59,2)	172(80,4)	<10^−5^
Métallique + plastique	10(2,3)	4(1,8)	6(2,8)	0,5

+ Résultats représentés sous forme N(%), SCN = Staphylocoque à coagulase négative, E Coli= Escherichia coli

La distribution des isolats en fonction de la nature du service hospitalier était: services médicaux (56,5%), laboratoires (29,6%) et services chirurgicaux (13,9%) avec une différence qui n’était pas statistiquement significative(p = 0,179) (Tableau 2). La répartition des isolats selon la fonction des personnels de santé a montré la prédominance des médecins (110/223 = 49,3%) suivi des infirmiers (43/223 = 19,3%), aides soignants (19/223 = 8,5%), techniciens de laboratoire (26/223 = 11,7%), pharmaciens (12/223 = 5,4%), secrétaires médicaux (10/223 = 4,5%) et ingénieurs(3/223 = 1,3%) avec une différence qui n’était pas statistiquement significative (p = 0,998) (Tableau 2). Les moyens de lavages des mains chez les personnels soignants étaient le savon (60/120 = 50%), solution hydro alcoolique (29/120 = 24%), savon associé à une solution hydro alcoolique (27/120 = 22%), eau (2/120 = 2%), povidone iodée(1/120 = 1%), savon+ povidone iodée (1/120 = 1%) et chez la population témoin les moyens de lavages des mains étaient le savon (116/120 = 97%) et savon associé à une solution hydro alcoolique (4/120 = 3%). La répartition de fréquence lavage de la main chez les personnels de santé était: moins de 5 fois par jour (22/120 = 18%), 5 à 10 fois par jour (88/120 = 73%) et plus de 10 fois par jour (11/120 = 9%) et dans la population témoin, elle était de moins de 5 fois par jour dans 6 cas (5%), 5 à 10 fois par jour dans cas 98 cas (82%) et plus de 10 fois par jour dans 16 cas (13%). La durée moyenne des téléphones était 19,22±13,364 mois chez les personnels de santé et 17,31± 13,572 mois (p = 0,148). Le nombre total de colonies des bactéries isolées était 113376 UFC avant la désinfection et 589 UFC après la désinfection. La désinfection des téléphones portables par la solution hydro alcoolique a réduit à 99,5% le nombre des colonies. Les taux de réduction après décontamination en fonction de la population étudié sont représentés dans le Tableau 3.

**Tableau 2:** Répartition des bactéries isolées des téléphones des personnels de santé en fonction des services, des fonctions et de la matière du couvercle du téléphone

		Germes isolés						total	p
			*SCN*	*Corynebactrium sp*	*S aureus*	*Bacillus sp*	*Autres*	N = 223	
			N = 113	N = 45	N = 49	N = 7	N = 9		
**Service**	services médicaux	N	62	30	26	2	6	126	0,174
		%	54,90%	66,70%	53,10%	28,60%	66,70%	56,5%	
	laboratoires	N	31	13	15	4	3	66	
		%	27,40%	28,90%	30,60%	57,10%	33,30%	29,6%	
	services chirurgicaux	N	20	2	8	1	0	31	
		%	17,70%	4,40%	16,30%	14,30%	0%	13,9%	
**Fonction**	médecins	N	55	22	24	4	5	110	0,998
		%	48,70%	48,90%	49,00%	57,10%	55,60%	49,3%	
	infirmiers	N	20	8	12	1	2	43	
		%	17,70%	17,80%	24,50%	14,30%	22,20%	19,3%	
	techniciens	N	12	6	5	1	2	26	
		%	10,60%	13,30%	10,20%	14,30%	22,20%	11,7%	
	ingénieurs	N	1	1	1	0	0	3	
		%	0,90%	2,20%	2,00%	0,00%	0,00%	1,30%	
	aides soignants	N	13	3	3	0	0	19	
		%	11,50%	6,70%	6,10%	0,00%	0,00%	8,50%	
	pharmaciens	N	6	2	3	1	0	12	
		%	5,30%	4,40%	6,10%	14,30%	0	5,40%	
	secrétaire médicaux	N	6	3	1	0	0	10	
		%	5,30%	6,70%	2,00%	0,00%	0,00%	4,50%	
	ingénieurs	N	1	1	1	0	0	3	
		%	0,90%	2,20%	2,00%	0,00%	0	1,30%	
**Matière du couvercle de téléphone**	métallique	N	43	18	21	2	3	87	0,937
		%	38,10%	40,00%	42,90%	28,60%	33,30%	39,0%	
	plastique	N	69	26	26	5	6	132	
		%	61,10%	57,80%	53,10%	71,40%	66,70%	59,2%	
	plastique + métallique	N	1	1	2	0	0	4	
		%	0,90%	2,20%	4,10%	0,00%	0,00%	1,80%	

SCN = Staphylocoque à coagulase négative, S aureus = Staphylococcus aureus

**Tableau 3:** Taux de réduction après décontamination des téléphones en fonction de la population étudiée

	Personnels de santé			Témoin		
Germes isolés	Nombre de colonies avant la désinfection (UFC)	Nombre de colonies après la désinfection (UFC)	Taux de réduction de colonies après la décontamination (%)	Nombre de colonies avant la désinfection (UFC)	Nombre de colonies après la désinfection (UFC)	Taux de réduction de colonies après la décontamination%
*SCN*	103241	302	99,7	4982	109	97,8
*Staph aureus*	3661	130	96,4	419	10	97,6
*Streptococcus sp*	23	0	100	-	-	-
*Corynebacterium sp*	417	26	93,8	494	0	100
*Bacillus sp*	12	0	100	9	0	100
*E.coli*	21	0	100	30	6	80
*Klebsiella sp*	-	-	-	1	0	100
*Pantoea sp*	10	0	100	-	-	-
*Acinetobacter sp*	-	-	-	10	5	50
*Candida sp*	26	1	96,1	20	0	100
Total	107411	459	99,6	5965	130	97,8

## Discussion

Nos résultats montrent que les téléphones mobiles constituent un réservoir des bactéries qui peuvent être associées aux infections communautaires ou nosocomiales. Dans notre étude, tous les téléphones étaient contaminés à 100% par des agents pathogènes. Ce taux était plus élevé que celui observé par d'autres chercheurs où le taux de contamination de téléphones mobiles des personnels de santé variait de 32% à 97,8% [2–4]. Ceci peut être expliqué par le manque d'hygiène des téléphones et des mains. Donc, il faut conseiller et sensibiliser à tous le monde d'effectuer la décontamination des téléphones mobiles. Nos données témoignent la prédominance des bactéries à Gram positifs: les cocci à Gram positif et les bacilles à Gram positif représentaient 76,7% et 21,1% des microorganismes isolés respectivement, tandis que les bacilles à Gram négatif ne représentaient que 1,3%. Les cocci à Gram positif et les bacilles à Gram positif font partie des flores commensales de la peau et des muqueuses chez l'homme et peuvent également se retrouver dans l'environnement [5]. Les bacilles à Gram négatif ne résident pas sur l'environnement sec de la peau normale. Occasionnellement, les surfaces intertrigineuses humides permettent la croissance d’*Acinetobacter sp* 5. Ces résultats témoignent que les bactéries contaminant les téléphones mobiles font partie de la flore cutanée. Ceci a été démontré par d'autres chercheurs [3]. Des études antérieures sur la contamination bactérienne des téléphones mobiles ont montré que les téléphones mobiles étaient contaminés par les isolats de SCN, *Bacillus spp, S. aureus, Escherichia coli, Klebsiella pneumoniae, Acinetobacter sp, Enterococcus faecalis et Pseudomonas aeruginosa, Pseudomonas fluorescensis* 4. Le profil des micro-organismes isolés dans notre étude est similaire à celui des autres études. La différence des taux d'isolement entre les bactéries contaminant les téléphones des personnels de santé et celle des téléphones de la population témoin n’était pas statistiquement significative sauf pour SCN et S aureus. Dans notre étude, Les souches de SCN étaient fréquemment isolées (57,7%) suivi de *Corynebacterium sp* (18,8%), *Staphylococcus aureu*s (18,1%) et *Bacillus sp* (2,3%). La prédominance des isolats de Staphylococccus sp contaminant les téléphones des personnels de santé a été retrouvé dans les autres études. Dans une étude réalisée en Turquie en 2007, les isolats SCN représentaient 68% des isolats [6], une autre étude réalisée en 2009 en Turquie a montré la prédominance de *S aureus*(52%) [7] et une étude nigérienne les isolats de *Staphylococcus epidermidis* étaient plus fréquents (42.9%) [3]. Notre étude a démontré qu'il n'y avait pas de différence significative entre la prévalence des bactéries isolées selon les services cliniques et la fonction des personnels de santé mais la différence de taux d'isolement des bactéries en fonction de la nature du couvercle du téléphone entre les personnels de santé et la population témoin était statistiquement significative sauf pour les couvercles mixtes (métallique + plastique). Les cultures des bactéries isolées au niveau des téléphones mobiles du personnel médical étaient plus polymorphes que celles de la population témoin, ceci prouve que les téléphones du personnel médical sont contaminés par les différentes espèces bactériennes. Les téléphones mobiles sont souvent en contact avec les surfaces contaminés et sont conservés dans des sacs à main et dans poches des utilisateurs ce qui expliquerait la présence de deux ou plus de deux espèces de bactéries sur les téléphones. Dans une étude réalisée en Inde par Sham et al en 2011 a démontré que les cultures polymicrobiennes ont été détectées dans 17,7% des téléphones mobiles des médecins dentistes et dans 388% des téléphones des médecins [8]. Dans le milieu hospitalier, les micro-organismes peuvent être transférés de personne à personne ou de l'environnement aux personnes et vice versa. Quelques études ont démontré que certains outils qui sont couramment utilisés et qui sont le plus souvent en contact avec la main tels que les stéthoscopes, les thermomètres, les claviers et les écrans des ordinateurs, les stylos, les dossiers des patients, les téléphones mobiles et les téléphones fixe, jouent un rôle essentiel dans la transmission des infections nosocomiale [9–11] Certaines études ont rapportés que les micro-organismes isolés à partir de téléphones mobiles de personnels de santé étaient similaires à ceux colonisant leurs mains [7]. L'hygiène des mains est un élément essentiel dans la lutte contre les infections associées aux soins.

La fréquence de lavage des mains chez la plupart de ces deux populations était entre 5 à 10 fois par jour. Malgré l'utilisation des désinfectants par certains personnels de santé dans les lavages des mains, il n'y avait pas de différence statistiquement significative de nombre de colonie entre ces deux populations. Le lavage des mains était largement dominé par le savon chez les témoins (97%). La moitié du personnel de santé utilisait le savon comme seul moyen de lavage des mains, le reste de la population employait la solution hydroalcoolique, povidone iodée, l'association savon- solution hydroalcoolique et savon-povidone iodée. Ces résultats témoignent que les moyens de lavage des mains utilisées par la population étudiée ne sont pas efficaces car certaines études ont confirmées qu'après le lavage des mains au savon, la flore transitoire restait sur les mains mais cette flore disparaissait en cas de traitement des mains par une solution hydroalcoolique [12, 13]. D'autres publications ont témoigné aussi l'efficacité de l'utilisation des solutions hydroalcooliques par rapport aux savons doux lors de lavage des bijoux [14] ou de faux ongles [15]. La durée de lavage des mains aussi est importante, 30 secondes permettant l’élimination de la flore transitoire, mais pas cinq secondes [16] et il a été prouvé que l'utilisation d'un volume de 3 ml de la solution hydroalcoolique permettait d'empêcher la persistance d'une flore bactérienne plus importante sur la main qu'un volume de 1ml de cette solution [17]. La désinfection des couvercles téléphoniques par une solution hydroalcoolique a réduit à 99,5% le nombre de colonie. Elle était active sur la totalité des bactéries isolées, avec un taux de réduction de colonie après la décontamination qui est légèrement élevée chez les personnels de santé (99,6%) par rapport à la population témoin (97,8%). Ceci témoigne l'efficacité de ce désinfectant sur le portage bactérien. La solution hydroalcoolique constitue le meilleur moyen de décontamination de téléphones mobiles en routine. Il est nécessaire d'introduire les recommandations concernant la décontamination des téléphones mobiles dans notre hôpital et à l'extérieur de l'hôpital. L'introduction des solutions hydroalcooliques en milieu hospitalier représente indiscutablement un progrès permettant d'améliorer l'observance de l'hygiène des mains par le personnel en lui apportant une méthode simple, rapide et efficace [18]. Leur intérêt et leur efficacité par rapport à la désinfection des téléphones portables doivent être bien expliqués au personnel. Les actions de formation doivent être répétées et toucher l'ensemble du personnel. La mise à disposition doit être complète, en s'assurant que les solutions hydroalcooliques sont effectivement disponibles au plus près des actes de soins, à portée de main immédiate des soignants. Il est également nécessaire de s'assurer de la coopération des cadres médicaux et infirmiers des services, qui jouent un rôle moteur dans l'application de ces mesures. C'est le rôle des équipes opérationnelles d'hygiène de diffuser une information claire à cet égard, et de s'assurer que ces conditions sont remplies [19]. La formation du personnel de santé sur le contrôle des infections, l'hygiène hospitalière, la désinfection de l'environnement, est d'une grande importance [20], et puisque les restrictions sur l'utilisation des téléphones portables par les personnels de la santé dans les hôpitaux n'est pas une solution pratique, ces derniers doivent être informés que ces dispositifs peuvent être une source de transmission des infections nosocomiales.

## Conclusion

Ce travail montre que les téléphones portables pourraient jouer un rôle dans la transmission des infections nosocomiales et communautaires. Il serait difficile d'interdire l'utilisation des téléphones mobiles au sein des services. C'est un moyen de communication entre les différents personnels de santé, mais nous pourrions facilement éviter la propagation des infections bactériennes en utilisant simplement les agents de nettoyage régulier et en réorganisant notre environnement. Dans ce cadre, il faut sensibiliser les utilisateurs des téléphones mobiles sur l'importance du lavage des mains et l'utilisation des solutions hydro alcoolique pour désinfecter aussi bien les téléphones portables que les mains.
